# Exploration of Attentional and Executive Abilities in French-Speaking Children Immersed in Dutch Since 1, 2, 3, and 6 Years

**DOI:** 10.3389/fpsyg.2020.587574

**Published:** 2020-12-17

**Authors:** Sophie Gillet, Cristina Anca Barbu, Martine Poncelet

**Affiliations:** Psychology and Neuroscience of Cognition Research Unit, University of Liège, Liège, Belgium

**Keywords:** second-language immersion education, cognitive advantage, attention, executive function, cognitive flexibility, working memory

## Abstract

Advantages in diverse aspects of cognitive functioning have been reported in early bilinguals (Bialystok, 2011) as well as in children frequenting an early bilingual immersion school program (Nicolay and Poncelet, 2015). However, during the last decade, some studies failed to replicate these advantages. Currently, the presence of cognitive benefits in children frequenting an immersion program remains debated. The lack of consistency between the studies could come from the fact that time spent by children within the immersion program is variable from one study to the other and that studies used different tasks to assess the same cognitive function. The main aim of the present study was to determine how time spent in immersion affects the emergence of cognitive advantages along the primary schooling. We compared 196 immersed Dutch-speaking children since they were 5 years old and 195 non-immersed French-speaking children, from different grades of the primary schooling (i.e., at 6, 7, 8, and 12 years old) by using the same attentional and executive tasks as those used in previous studies having shown a bilingual advantage. Furthermore, these groups were matched on a set of variables known to influence cognitive functioning. After 1, 2, and 3 years of enrolment in this program, performances of immersed compared to non-immersed children did not differ for any task. However, after 6 years, immersed children outperformed non-immersed children on the cognitive flexibility and the working memory tasks. These results show that, in French-speaking children immersed in Dutch, cognitive advantages could depend on the length of time spent in immersion since they are not present at the beginning (after 1, 2, and 3 years) but seem to emerge at the end of it (after 6 years). In contrast, in previous studies conducted in English immersion, advantages appear at the beginning of the primary schooling but are absent at the end of it. Furthermore, these results suggest that the emergence of cognitive advantages may vary depending on the second language learned. The results are discussed in terms of linguistic characteristics and status of the languages at stake.

## Introduction

A large number of studies have shown that early bilingualism can positively affect cognitive functions such as inhibition and cognitive flexibility (Bialystok et al., 2004; Bialystok et al., 2008; Costa et al., 2008; Adesope et al., 2010; Prior and MacWhinney, 2010; Antón et al., 2019), attentional abilities (e.g., Chung-Fat-Yim et al., 2016), and working memory (e.g., Blom et al., 2014). These advantages were also revealed in immersion education programs such as the Content and Language Integrated Learning (CLIL) program where children are exposed to a second language (L2) early (for example, as soon as third kindergarten) and massively, between 50 and 75% of school time. A key characteristic of the CLIL program is that L2 is not taught as a foreign language but used to teach academic subjects in L2 by L2 native or native-like teachers (Comblain and Rondal, 2001). CLIL exists in different countries but the manners to organize it are multiple (e.g., balance between language and content instruction, instructional goals, pedagogical approaches to integrating language and content instruction which can differ) (for more information, see Cenoz et al., 2014).

As for early bilingualism at home, cognitive advantages have been shown in CLIL contexts. The challenging CLIL context, in which children are learning the subjects in an L2, has been shown to enhance their attentional and executive abilities (e.g., Nicolay and Poncelet, 2013a, 2015; Hansen et al., 2016). According to these authors, attentional and executive processes like alerting, selective attention, divided attention, cognitive flexibility and working memory are highly required and trained when learning an L2 in a CLIL context. Alerting skills refers to the capacity to quickly react to stimuli and are supposed to be particularly recruited in CLIL to hold a continuous readiness state to process an L2. Selective attention skills refers to the ability to select the pertinent information and to inhibit other non-pertinent information. This function is supposed to be highly required in CLIL in order to understand and treat L2 linguistic input in which the child is not yet fluent. Divided attention skills permit to share attention between different stimuli and, for example, would permit to simultaneously treat L2 auditory and visual information presented in class (Barbu et al., 2019). Cognitive flexibility refers to the ability to be flexible enough to adjust to changed demands or unexpected opportunities while working memory refers to holding information in mind and mentally working with it (Diamond, 2012). Cognitive flexibility is supposed to be highly solicited to alternate between the linguistic contexts (L1 or L2 classes) while working memory could be highly solicited to maintain L2 information the time to understand or to infer the meaning of the sentence heard. Consequently, as previously suggested by other studies (Nicolay and Poncelet, 2013a, 2015; Barbu et al., 2019), these different attentional and executive functions might be highly required and trained and thus, develop faster in children frequenting the CLIL context.

However, some studies failed to replicate the attentional and executive advantage in both immersed children (e.g., Kaushanskaya et al., 2014; Simonis et al., 2019) and early bilinguals (cf. the meta-analysis of Paap et al., 2015). In the CLIL literature focusing on cognitive advantages in primary CLIL schooling, only about half of the studies showed advantages in attentional and/or executive functions for immersed children compared to non-immersed children (Nicolay and Poncelet, 2013a, 2015; Kaushanskaya et al., 2014; Kalashnikova and Mattock, 2014; Puric et al., 2017; Barbu et al., 2019). Different factors could explain these inconsistent results. Firstly, the time spent in immersion could influence the outcomes. Bialystok and Barac (2012) showed that the time spent in immersion was related to performance on executive control tasks. In their study, performance improved with increasing experience in the immersive environment. Secondly, the tasks used to evaluate attentional and executive functions (AEF) could also influence the outcomes. A recent meta-analysis (Ware et al., 2020) showed that the type of task used to assess executive functioning influenced the magnitude of the difference between bilinguals and monolinguals. For example, bilingual advantage is consistently observed on the Attentional Network Task but not on the Flanker Task despite the fact that these two commonly used tasks are highly similar and are supposed to evaluate the same AEF processes. The similarity between the languages at stake could also be a potential confounding factor explaining the inconsistency. As in bilinguals both languages are constantly activated (Kroll et al., 2012), this parallel activation leads to bidirectional cross-language interactions that have to be controlled cognitively in function of the language context. These cross-language interactions could differ depending on the similarity between the two languages spoken and could vary through the different linguistic levels (i.e., phonological, lexical, syntactic…) (Oschwald et al., 2018). Consequently, language similarity could affect the amount of attentional and executive control required to use L1 and L2 effectively (Barac and Bialystok, 2012; Coderre and van Heuven, 2014). Another factor concerns the matching of the immersed and non-immersed groups on different control variables. For example, some studies did not match the groups on socioeconomical status (SES). This is, for example, the case of the study of Kalashnikova and Mattock (2014). However, Noble et al. (2005) showed that parental education and parental occupation (which is an index of SES) were responsible for more than 14% of the variance in the outcomes in executive function tests in children. Different other activities have also been shown to influence attentional and executive functioning like video games (Choi et al., 2020), sport practice (for a review, see Diamond and Lee, 2011) and music training (Janus et al., 2016) as well as some particular school curricula (e.g., Tools of the Mind or Montessori) (Diamond, 2012).

These factors are rarely all controlled among the CLIL studies conducting until now. Video game, sport, and music practice, are variables that were not taken into account to match the groups in any study, except in Barbu et al. (2019). Moreover, among the studies, combinations of different factors such as the number of years spent in immersion, which vary from one study to another, the tasks used to evaluate attentional and executive functions (AEF), the L1-L2 combination studied, and the different CLIL contexts, could have interacted with each other, which makes the interpretation of the outcomes difficult. For example, Kalashnikova and Mattock (2014) showed an advantage after approximately one year (from 0.7 years to 1.7 years) of immersion on cognitive flexibility. In another study (Puric et al., 2017), also evaluating children immersed since one year, no difference in cognitive flexibility was found between the groups. However, in the first study, English-speaking children were learning Welsh as a second language and the task used to evaluate cognitive flexibility was the dimensional change card sort (DCCS; Frye et al., 1995). Meanwhile, in the other study, Serbian-speaking children were learning English or German, and the tasks used to evaluate cognitive flexibility were a local-global task adapted from Huizinga et al. (2006) and a color-shape task developed by Puric et al. (2017). Moreover, in these two studies, the CLIL context was dissimilar. The children of the first study began the immersion program at approximately 4 years old and those of the second one, at 7 years old. Finally, the two studies did not use the same variables to match the groups. The first study matched the groups on age and receptive vocabulary, while the second study matched the groups on age, intelligence, and SES. To resume, in these two studies, the CLIL contexts were dissimilar and the tasks used to evaluate cognitive flexibility were different. Moreover, the two languages at stake were different, and the controlled variables used to match the groups differed. All these differences render the interpretation of the results difficult. Table 1 comprises the studies conducted to evaluate cognitive development in immersed children in primary school. This table highlights the variability of findings as well as the differences observable between studies in terms, for instance, of tasks used to assess AEF or ages of starting the immersion program. These differences, from one study to the other, could modulate the outcomes. These studies were classified by the number of years spent within the immersion program (from the shortest to the longest). For each study, the following information, if available, is presented: number of participants, time participants spent within the immersion program, specific L1 and L2 languages, age when starting the immersion program, the function targeted by the tasks, the attentional and executive tasks used, data about the presence of advantages for immersed children, and finally, the variables controlled. The other functions evaluated in the studies have also been mentioned but we focused on attentional and executive functions.

**TABLE 1:** Studies conducted to evaluate cognitive development in immersed children classified by number of years of immersion context experience, the age of starting immersion, the L1/L2 pair implicated, the cognitive functions targeted, the tasks used, the presence of cognitive advantages and the variables controlled.

**Study**	**Participants (age at the moment of the study)**	**Time spent in immersion**	**L1**	**L2**	**Age when starting immersion**	**Cognitive function targeted**	**Attentional and executive tasks (including working memory)**	**Cognitive advantage? + comments**	**Variables controlled**
Carlson and Meltzoff (2008)	21 immersed	0.5 years	English	Spanish (n = 13) or Japanese (n = 8)	5 years	Inhibition	9 measures:	NO for immersed children	Age
	(5;10 years) 17 monolinguals (6;3 years) + bilinguals					Cognitive flexibility Visual short term memory Non-verbal reasoning	Simon Says, Reflection/impulsivity Scale, Delay of gratification, Statue, Gift Delay with cover, Attention Network Task (ANT), Advanced DCCS, Visual cued recall Kansas, C-TONI		Expressive vocabulary SES via ANCOVA

Kalashnikova and Mattock (2014)	33 immersed (4;6 years) 33 monolinguals (4;6 years)	Group nursery classes	English	Welsh	No available (na)	Cognitive flexibility	DCCS	YES	Age
		0.7 years				Metalinguistic awareness Meta-representational ability	Moving word task Appearance/reality task	NO NO	Receptive vocabulary
		Group receptive classes 1.7 years						The children live in a bilingual community	

Woumans et al. (2016)	27 immersed	1 year	French	Dutch	5 years	Inhibition	ANT	NO	Age
	(5;2 years)						Simon task	NO	Gender
	27 monolinguals					Verbal flexibility	Verbal fluency task	NO	Intelligence SES
	(5;3 years)					Non-verbal analytic reasoning	Raven’s Colored Matrices	YES	Verbal
									Fluency
									Cognitive control
									Paired at T0

Puric et al. (2017)	19 immersed high exposure (HE; 7;9 years)	1 year	Serbian	English or German (HE)	7 years	Inhibition Cognitive flexibility	Non-verbal Stroop task Local-global task Color-shape task	NO NO NO	Age Intelligence SES
	17 immersed low exposure (LE; 8; 2 years) 22 monolinguals (7;10 years)			French (LE)		Working memory	Counting recall task Backward digit span task	YES (HE) YES (HE)	
Barbu et al. (2019)	59 immersed	1 year	French	English	5 years	Cognitive flexibility	Cognitive flexibility task	NO	Age
	(6;7 years)					Alerting	Alerting task	NO	Gender
	57 monolinguals (6;8 years)					Selective auditory attention	Selective attention task	YES	Intelligence SES
						Divided attention	Divided attention task	NO	Receptive vocabulary
									Extra scholar activities

Gillet et al. (in press)	196 immersed and 195 monolinguals (from 6 to 12 years old)	1, 2, 3, and 6 years	French	Dutch	5 years	Cognitive flexibility	Cognitive flexibility task	YES (6th grade)	Age
						Working memory	Backward digit span task	YES (6th grade)	Gender
						Alerting	Alerting task	NO	Intelligence
						Selective auditory attention	Selective attention task	NO	SES
						Divided attention	Divided attention task	NO	Receptive vocabulary
									Extra scholar activities

Kaushanskaya et al. (2014)	19 immersed (6;7 years) 19 monolinguals (6;3 years)	1.96 years of dual immersion classroom experience, with a range of 0.25 years to 4.17 years.	English	Spanish	5 years	Cognitive flexibility Working memory Word learning	DCCS Listening-span task Word-span task Two word learning tasks (novel words mapping with familiar or non-familiar referents)	NO YES NO YES (only for familiar referents)	Age Intelligence SES Receptive vocabulary

Poarch and van Hell (2012)	19 immersed (6;9 years) 18 bilinguals (6;8 years) 18 trilinguals (6;8 years) 20 monolinguals (7;1 years)	Experiment 1:1.3 years Experiment 2: ± 2 years	German	English	5 years	Inhibition Inhibition	Experiment 1: Simon task Experiment 2: ANT	NO advantage for immersed children No comparison with monolinguals	Age SES Receptive vocabulary
Poarch and Bialystok (2015)	203 children (8;11 years old) Monolinguals (9;5 years) Partially bilinguals (9;7 years) + bilinguals and trilinguals	2 years	English	French	± 7 years for the partially bilinguals	Inhibition	Modified Flanker task	NO (for partially bilingual/immersed children)	Age Intelligence SES Receptive vocabulary

Hansen et al. (2016)	76 immersed and 76 monolinguals (7 through 14 years old)	2 to 8 years	Spanish	English	6 years	Working memory	N-back (update WM)	YES in Grade 2 and 3 No difference in Grade 5 or 8	Age, Gender, SES, Fluid intelligence, Home literacy education

Nicolay and Poncelet (2013a)	53 immersed (8;7 years) 51 monolinguals (8;5 years)	3 years	French	English	5 years	Inhibition of interference	ANT	NO	Age
						Response inhibition	Response Inhibition task	NO	Gender
						Cognitive flexibility	Cognitive flexibility task	YES	Intelligence
						Alerting	Alerting task	YES	SES
						Selective auditory attention	Selective attention task	YES	Receptive vocabulary
						Divided attention	Divided attention task	YES	

Nicolay and Poncelet (2015)	51 immersed	3 years	French	English	5 years	Response inhibition	Response inhibition	NO	Age
	(5;3 years)					Cognitive flexibility	Cognitive flexibility	YES	Gender
	50 monolinguals					Alerting	Alerting task	YES	Intelligence
	(5;4 years)					Selective auditory attention	Selective attention task	YES	SES
						Divided attention	Divided attention	YES	Receptive vocabulary
								Follow-up study	Paired at T0
Bialystok and Barac (2012)	Study 1: 100 children (8;2 years, grade 2 and 3) Study 2: 80 children (7;8 years, grade 2 and 10;7 years, grade 5)	Variable (3-4-5 years)	Study 1: English and/or Hebrew or Russian Study 2: English or English + another language	Study 1: Hebrew Study 2: French	Children can enter at any time, not controlled	Inhibition Cognitive flexibility Metalinguistic awareness Cognitive flexibility Metalinguistic awareness	Study 1: Flanker task Task switching Metalinguistic task Study 2: Task switching Metalinguistic task	EF increased with immersion experience Metalinguistic abilities with L2 level	

Simonis et al. (2019)	255 immersed (primary and secondary) 258 monolinguals (primary and secondary) (10 and 16 years old)	5 years (primary) or 12 years (secondary)	French	Dutch or English	5 years	Inhibition	Simon task	NO	Age
							ANT	NO	Intelligence
						Flexibility	DCCS	NO	Gender
								In primary and secondary	SES
									Bilingualism
									Receptive vocabulary
									Via ANCOVA

Simonis (2019)	318 immersed (primary and secondary) 330 non-immersed (11 and 17 years old)	6 years (primary) or 12 years (secondary)	French	Dutch or English	5 years	Auditory sustained attention	Auditory Sustained Attention task	NO	Age
						Auditory selective attention	Auditory selective attention task	YES	Intelligence
						Visual selective attention	Visual Selective Attention task	NO	Gender
						Divided attention	Divided Attention task	NO	SES
								NO	Bilingualism
									Receptive vocabulary
									Via ANCOVA

Nicolay (2012)	37 immersed and 37 non-immersed (11-12 years old)	6 years	French	English	5 years	Inhibition of interference	ANT	NO	Age
						Cognitive flexibility	Cognitive flexibility task	NO	Intelligence
						Alerting	Alerting task	NO	SES
						Divided attention	Divided attention task	NO	Receptive vocabulary (French)

*Note. EF, Executive functions; T0, Time 0 before starting immersion.*

The variability highlighted in this table could partly be due to the fact that the data was collected in different countries which all have different interpretation of CLIL. A certain number has nevertheless adopted a similar methodology focusing on a similar CLIL context to evaluate AEF in immersed children. These studies were conducted in the French-speaking part of Belgium where all the schools organize CLIL following the same model, with either English, Dutch, or German as L2. These three languages are all Germanic languages and share many properties and constructions by virtue of common ancestry (for more details, see Harbert, 2006). The immersed children start immersion at the same moment, that is to say, in third kindergarten, at 5 years old. All the children are immersed from 50 to 75% of their school time in their L2 and the program content is the same for all the CLIL schools. In addition, with the exception of second language learning, the program content is similar in CLIL and non-CLIL schools and lead to the same basic study certificate. Until now, most of the studies conducted in Belgium evaluating CLIL impact on AEF focused on English as L2 (Nicolay, 2012; Nicolay and Poncelet, 2013a, 2015; Barbu et al., 2019; Simonis, 2019; Simonis et al., 2019) and few focused on Dutch as L2 (Woumans et al., 2016; Simonis et al., 2019). No study focused on German to our knowledge. Among the studies conducted in Belgium using the same tasks when assessing AEF, the results of the studies conducted in English are the following ones.

In *first grade*, Barbu et al. (2019) found an advantage in a selective auditory attention task but not in alerting, divided attention and cognitive flexibility in children immersed in English. In *third grade*, Nicolay and Poncelet (2013a, 2015) found advantages in alerting, selective auditory attention, divided attention, and cognitive flexibility but not in inhibition in children immersed in English. Finally, in *sixth grade*, Nicolay (2012) found no difference in the same tasks as that used in studies evaluating children in first and third grades (Nicolay and Poncelet, 2013a, 2015; Barbu et al., 2019) in immersed children learning English. Simonis (2019) further did not find advantages in sixth grade on tasks measuring auditory sustained attention, auditory selective attention, visual selective attention, and divided attention. In Dutch, one study has been conducted on AEF in CLIL context with the same tasks of the present experiment. In *sixth grade* (Simonis, 2019) no advantage in a group of Dutch immersed children mixed with English immersed children was found.

To resume, some advantages are observable at the beginning of the CLIL program in first- and third-grade children learning English, which suggests that their abilities could have been boosted by immersion. However, these advantages are no longer observable at the end of the CLIL program in English and Dutch. This is in contradiction with previous studies. Indeed, as a reminder, Bialystok and Barac (2012) showed that time spent in the CLIL context was linked to enhanced executive functions. Consequently, we should expect to observe AEF advantages more likely in studies evaluating children at the end of the CLIL schooling. These data suggest that the cognitive advantages highlighted, at least, in English immersed children, are not necessarily sustainable. One explanation advanced for this was that non-immersed children filled the gap during normal cognitive development (Nicolay, 2012; Simonis, 2019). However, more studies should be conducted to confirm these findings.

### The Present Study

The main aim of the present study was to evaluate when AEF advantages emerge in a CLIL context in Dutch immersed children using the same tasks as previous studies showing an advantage. Therefore, we evaluate the children at different moment of the schooling, i.e., in first, second, third, and sixth grade (that is to say respectively at 6, 7, 8 and 12 years of age and after 1, 2, 3, and 6 fully accomplished years of immersion). We controlled for a maximum of variables likely to modulate the emergence of cognitive advantage, that is to say, SES (e.g., Mezzacappa, 2004; Ardila et al., 2005; Hughes and Ensor, 2005; Noble et al., 2005), gender (Huster et al., 2011), L1 lexical level and non-verbal reasoning (Morton and Harper, 2007; Li and Xie, 2017; Czapka et al., 2020), sport, music, or video game practice (for a review, see Diamond, 2012). Only the study of Barbu et al. (2019) controlled for the video game, sport, and music practice of the children in addition to the factors that were often, but not always, controlled in the other studies (SES, gender, time spent in CLIL, L1 lexical level and non-verbal reasoning). Therefore, if we obtain a cognitive advantage in immersed children, we could not attribute it to any known confounding factors. This controlled matching would allow establishing if a real advantage of L2 immersion exposition exists, as some researchers doubt the existence of a bilingual cognitive advantage (Paap et al., 2015).

The participants were evaluated using the same attentional and executive tasks as in previous studies, showing an advantage in selective auditory attention for immersed children in English in first grade (Barbu et al., 2019) and in alerting, selective auditory attention, divided attention and cognitive flexibility in third grade (Nicolay and Poncelet, 2013a, 2015). The CLIL program in which the children of the present study were integrated is also highly similar to those of the studies of Nicolay and Poncelet (2013a, 2015); Woumans et al. (2016); Barbu et al. (2019) and Simonis (2019). The children are immersed at 50 to 75% of their school time in their L2 since they are 5 years old and follow a same content program (which is the same as for non-immersed children). A task measuring working memory was added to the protocol used by the previous studies (Nicolay and Poncelet, 2013a, 2015; Barbu et al., 2019) as some studies showed that this skill could also be enhanced by CLIL context (Kaushanskaya et al., 2014; Hansen et al., 2016; Puric et al., 2017) and is very important in order to achieve academic success (Diamond, 2012). Finally, in addition to the attentional and executive abilities assessed, we evaluated the Dutch lexical skills of the immersed children to determine the level of L2 acquired in the CLIL context.

The children tested during the present study frequented a highly similar CLIL context as compared to the English-immersed children tested by Nicolay (2012); Nicolay and Poncelet (2013a, 2015); Barbu et al. (2019); and Simonis (2019). This CLIL solicitation should trigger the same AEF advantages on the children of the present study. Moreover, Dutch and English are both Germanic languages. Consequently, based on previous findings, we expected an advantage on selective auditory attention in first grade but not on the other functions (except for working memory). Given that we additionally assessed working memory, we also expected an advantage at this level right from the first years of immersion (e.g., Hansen et al., 2016). To our knowledge, working memory has, not yet been studied in this CLIL context with a French-English (or Dutch) pair. A greater number of cognitive functions would be enhanced in second grade and as children are longer exposed to their L2, we expected a CLIL advantage on all the functions in third grade (Nicolay and Poncelet, 2013a, 2015). Also according to previous studies conducted in English, no advantages would be present in sixth grade for immersed children, as monolinguals would have filled the gap with normal cognitive development (Nicolay, 2012; Simonis, 2019).

## Method

### Participants

Three hundred ninety-one typically developing French-speaking children of primary schooling took part in the study. The sample included 106 children in first grade (53 immersed and 53 non-immersed), 108 children in second grade (53 immersed and 55 non-immersed), 99 children in third grade (51 immersed and 48 non-immersed), and 78 children in sixth grade (39 immersed and 39 non-immersed). The participants were recruited from immersion and traditional schools in the French-speaking part of Belgium. The sample characteristics are presented in Table 2, indicating descriptive statistics and mean comparisons for age, a non-verbal intelligence measure, and French receptive vocabulary for each grade, and in Table 3, describing the descriptive statistics and comparisons for gender, SES, and extra-scholar activities for each grade. The criteria of inclusion in the study were that the children of the two groups were native speakers of French, had not repeated or skipped grades, did not suffer from neurological disorders or sensory deficits, and presented no history of speech or language impairment. Children speaking two languages at home or in their family, or following extra-scholar lessons in a second language were excluded from the sample. Concerning second language learning, note that in the French-speaking community of Belgium, children attending a traditional education receive L2 instruction (in English or Dutch) starting from fifth grade and at the rate of two hours a week.

**TABLE 2:** Descriptive statistics and mean comparisons for age, non-verbal intelligence (Raven), and French receptive vocabulary (EVIP) for each grade (*N* = 391).

	**Grade 1**		**Grade 2**		**Grade 3**		**Grade 6**	
	**I (*n* = 53) Mean (SD)**	**NI (*n* = 53) Mean (SD)**	**I (*n* = 53) Mean (SD)**	**NI (*n* = 55) Mean (SD)**	**I (*n* = 51) Mean (SD)**	**NI (*n* = 48) Mean (SD)**	**I (*n* = 39) Mean (SD)**	**NI (*n* = 39) Mean (SD)**
Age *in months*	79 (3)	80 (3)	91 (3)	92 (3)	105 (4)	105 (4)	139 (3)	140 (4)
*t*-test value, *p*	1.19 0.23		0.94 0.34		−0.02 *0.98*		0.91 *0.98*	
Raven CR, max 36 and 60 in sixth grade	22.2 (3.7)	22.5 (4.3)	23.6 (4.5)	24.1 (3.9)	27.4 (3.6)	26.3 (4.3)	43.3 (3.9)	41.4 (5.4)
*t*-test value, *p*	0.42 0.66		−0.62 0.53		1.38 0.16		1.67 0.10	
EVIP CR, max 170	86.5 (13.3)	85.8 (15.8)	91.0 (14.7)	90.8 (14.1)	107.3 (16.2)	106.6 (16.4)	135.4 (8.1)	135.5 (12.7)
*t*-test value, *p*	−0.25 *0.80*		0.07 0.93		0.23 0.81		−0.06 0.94	

*NI, Non-Immersed; I, Immersed; SD, Standard Deviation; CR, Correct Response.*

**TABLE 3:** Descriptive statistics and comparisons for gender, SES, and extra-scholar activities for each grade.

	**Grade 1**		**Grade 2**		**Grade 3**		**Grade 6**	
	**I (*n* = 53)**	**NI (*n* = 53)**	**I (*n* = 53)**	**NI (*n* = 55)**	**I (*n* = 51)**	**NI (*n* = 48)**	**I (*n* = 39)**	**NI (*n* = 39)**
Ratio (m:f)	24:29	25:28	29:24	24:31	24:27	22:26	13:26	24:15
Test Chi^2^, *p*	X^2^ (1) = 0.03, 0.84		X^2^ (1) = 1.32, 0.24		X^2^ (1) = 0.01, *0.90*		X^2^ (1) = 6.22, *0.01*	
Sociocultural level^a^ 1 2 3 4	0 13 23 17	1 24 14 14	0 13 24 16	0 17 18 20	0 15 23 13	3 21 15 9	1 6 19 13	1 14 14 10
Test Chi^2^, *p*	X^2^ (3) = 6.74, 0.08		X^2^ (2) = 1.79, 0.40		X^2^ (3) = 6.32, 0.09		X^2^ (3) = 4.34, 0.22	
Video practice^b^ 0 1 2 3 4	13 14 15 10 1	14 8 15 14 2	10 20 10 11 2	14 16 14 9 2	3 10 17 19 2	2 14 13 18 1	8 1 14 7 9	13 6 12 4 4
Test Chi^2^, *p*	X^2^ (4) = 3.33, 0.50		X^2^ (4) = 1.94, 0.78		X^2^ (4) = 1.67, 0.79		X^2^ (4) = 7.67, 0.10	
Sport practice^b^ 0 1 2 3 4	4 4 10 27 8	7 0 7 27 12	5 2 19 23 4	5 2 14 26 8	0 2 13 21 15	5 3 13 12 15	1 5 18 11 4	0 2 13 15 9
Test Chi^2^, *p*	X^2^ (4) = 6.52, 0.16		X^2^ (4) = 2.23, 0.67		X^2^ (4) = 7.94, 0.15		X^2^ (4) = 5.63, 0.22	
Music practice^b^ 0 1 2 3 4	41 1 7 4 0	46 0 2 6 0	40 4 3 2 1	45 4 3 2 1	43 4 0 4 0	39 2 2 4 1	32 3 4 0 0	28 4 6 1 0
Test Chi^2^, *p*	X^2^ (3) = 4.45, 0.21		X^2^ (4) = 0.05, 0.99		X^2^ (4) = 7.16, 0.12		X^2^ (3) = 1.81, 0.61	

*NI, Non-Immersed; I, Immersed. ^*a*^1, primary school level; 2, secondary school level; 3, high school level; 4, university level. ^*b*^0, no practice; 1, very little or little practice; 2, mean practice; 3, frequent practice; 4, very frequent practice.*

### Materials and Procedure

#### Background Measures

The two groups (immersed and non-immersed) within each grade (1, 2, 3, and 6) were matched on measures of age, socioeconomic status (SES), intellectual capacities and level of receptive vocabulary in the native language (French), as these factors may influence cognitive development (e.g., Morton and Harper, 2007; Huster et al., 2011). The groups were also matched on gender, except those in sixth grade, and in time spent on extra-scholar activities like sport, music, or video game practice, as these activities are likely to modulate executive functioning (for a review, see Diamond, 2012). Finally, none of the schools (immersion or non-immersion) used ‘active’ pedagogic curricula also known to improve executive functioning (Diamond, 2012).

A parental questionnaire provided us with data used to exclude some children and to match the groups, such as the level of education of the parents (used to determine the SES of the child), the precise age of the child, and the frequency of the child’s practice of different extra-scholar activities.

##### Socioeconomic status

We used the level of education of the parent that had the highest level as a proxy for socioeconomic status. Immersed and non-immersed children inside each grade were divided into four categories in terms of the higher diploma of their parents as reported on the questionnaire: 1 = primary; 2 = secondary; 3 = high degree; 4 = university degree.

##### Sport, music, or video game practice

To control for these extra-scholar activities, we asked the parents to evaluate the frequency of practice of their child, per week, on a 5-point Likert scale (0 = no practice; 1 = very little or little practice; 2 = mean practice; 3 = frequent practice; 4 = very frequent practice). The extra-scholar activities investigated were sport, music, and video game practice.

##### Non-verbal intelligence

Raven’s Progressive Matrices were administered to the participants to assess non-verbal reasoning abilities. Children from grades 1 to 3 were evaluated with the colored version of the test (Raven et al., 1998). The adult version was administered to the older children (sixth grade). The standardized procedure for each version was used for the administration and score calculation. Given that the immersed and non-immersed groups inside each grade were matched in terms of age, we used the raw score, which corresponds to the number of correct responses in the analyses.

##### Lexical receptive abilities in L1

The French adaptation of the Peabody Picture Vocabulary Test-Revised, the *Échelle de vocabulaire en images Peabody* (EVIP; Dunn et al., 1993), was used to evaluate the participants’ receptive vocabulary in L1. Children were required to select (by pointing at the right image) which of four line drawings corresponded to a word spoken by the experimenter. The standardized procedure was used for the score calculation. Given that the immersed and non-immersed groups inside each grade were matched on age, raw scores were used in the subsequent analyses.

#### Measures of Dutch Lexical Development

The level of L2 vocabulary knowledge was also evaluated to obtain an indication of the level attained by the immersed children after one, two, three, or six years of immersion.

Dutch vocabulary was evaluated by an adaptation of two experimental tasks (Nicolay and Poncelet, 2013b) used by Nicolay and Poncelet (2013a, 2015). These tasks were administered to properly assess the Dutch receptive and productive vocabulary knowledge learned in the particular context of immersion. Indeed, their L2 lexical development will be more school-dependent and less varied as a native speaker (Bialystok et al., 2010). Regarding the results and ceiling effects obtained by Nicolay and Poncelet (2013a) on these tasks in third grade, two standardized L2 vocabulary tests were administered in third and sixth grades. The task are described below and the results are presented in Table 4.

**TABLE 4:** Means (Standard Deviations) for the children’s version of the tasks administered to grades 1, 2, and 3 and the adult version of the tasks administered to grade 6.

	**Grade 1**		**Grade 2**		**Grade 3**		**Grade 6**	
	**I**	**NI**	**I**	**NI**	**I**	**NI**	**I**	**NI**
	**Mean (SD)**	**Mean (SD)**	**Mean (SD)**	**Mean (SD)**	**Mean (SD)**	**Mean (SD)**	**Mean (SD)**	**Mean (SD)**
Alerting								
Correct response	29.8 (0.7)	30 (0)	29.9 (0.1)	30 (0)	30 (0)	30 (0)	20 (0)	20 (0)
Median time in ms	392 (65)	380 (66)	352 (63)	358 (66)	339 (75)	331 (61)	258 (46)	256 (44)
Auditory attention								
Correct response	16.8 (2.7)	17.3 (2.1)	18.8 (1.2)	19.1 (1.3)	19.0 (1.1)	18.7 (2.2)	15.3 (1.4)	15.4 (0.93)
Median time in ms	869 (139)	876 (154)	773 (101)	774 (111)	768 (122)	735 (114)	628 (103)	642 (134)
Divided attention								
Correct response	32.4 (5.7)	33.6 (4.6)	36.5 (3.7)	36.0 (3.3)	36.6 (3.1)	35.9 (4.8)	15.1 (1.2)	14.8 (0.7)
Median time in ms	825 (95)	782 (111)	713 (86)	745 (102)	750 (99)	726 (105)	680 (147)	681 (129)
Cognitive flexibility								
Correct response	38.9 (5.7)	38.7 (6.4)	42.3 (4.4)	41.9 (4.1)	41.2 (4.3)	40.0 (7.1)	88.1 (8.3)	80.5 (10.9)
Median time in ms	1392 (266)	1273 (298)	1154 (258)	1104 (255)	1011 (203)	1006 (212)	907 (197)	954 (283)
Working memory								
Mean Span	3.1 (0.6)	3.1 (0.8)	3.6 (0.7)	3.5 (0.8)	3.6 (0.7)	3.6 (0.9)	4.6 (1.4)	3.9 (0.6)

##### Dutch productive vocabulary

In first and second grades, a Dutch productive vocabulary knowledge task (Nicolay and Poncelet, 2013b) used by Nicolay and Poncelet (2013a, 2015) and designed as a picture-naming task was used to directly probe the vocabulary learned at school. It consisted of a 135-item list based on the words that were supposed to be used at school during the first and second English immersion school years. We translated this task into Dutch for the present study. Children were asked to name the pictures in Dutch, and if they could not do so, at least in French to ensure that they had recognized the pictures and were familiar with the corresponding concepts. The total number of correct Dutch naming responses was scored for each child (maximum possible score = 135). Minor misarticulations were given full credit for a correct response, provided that they were sufficiently close to the target to be unambiguously identified as such.

In third and sixth grades, we used an adaptation of the Expressive One-Word Picture Vocabulary Test in English (Gardner, 2000), which we translated into Dutch. This test was administered to immersed children to evaluate their Dutch lexical production. The number of correct responses was used for each child.

##### Dutch receptive vocabulary

To assess the receptive vocabulary knowledge in Dutch acquired after 1, 2, or 3 years of immersion, an adapted English receptive vocabulary knowledge task translated into Dutch (Nicolay and Poncelet, 2013a) designed as a word-to-picture matching task was administered. The 135 items from the Dutch productive vocabulary task were used as well. They were distributed over 27 computer slides. Each slide contained five target pictures, to be pointed out one at a time, and two distractors. The total number of correct word-to-picture matching responses was scored for each child (maximum possible score = 135).

In third and sixth grades, we used the Dutch version of the Peabody Picture Vocabulary Test (PPVT-III-NL; Dunn et al., 2005) to evaluate receptive vocabulary. The number of correct responses was scored for each child. The receptive vocabulary raw score was transformed in lexical age acquisition to obtain an approximation of the Dutch receptive level attained by children attending an immersion school after 3 and 6 years.

#### Attentional and Executive Measures

We used tasks evaluating alerting, auditory selective attention, divided attention, and cognitive flexibility provided from standardized batteries (KITAP, Zimmermann et al., 2002: French adaptation by Zimmermann et al., 2005; TAP, Zimmermann and Fimm, 2010) and a task evaluating working memory (Wechsler, 2003). The Test of Attentional Performance has two versions: a child version for children from 6 to 10 years old (KITAP) and an adult version (TAP) for older children and adults. The children’s version was used for children from grades 1 to grade 3 and the adult version (TAP) was used for children in grade 6. The KITAP battery was also used by Nicolay and Poncelet (2013a, 2015) and Barbu et al. (2019) in their studies showing a CLIL advantage in children, respectively, in grade 3 and grade 1. The TAP battery was used by Nicolay (2012) and Simonis (2019) in their studies evaluating immersed children in sixth grade and in which no CLIL advantages were found.

##### Alerting

was measured using the KITAP task “*The Witch*” or the TAP task “*Alerting*”. In these tasks, a witch vs. a cross appeared in the middle of the computer screen. Children were asked to press a response key as fast as possible when the stimulus (a witch for the children version or a cross for the older) appeared. The number of correct responses and the median time reaction were used as dependent variables.

##### Auditory selective attention

Auditory selective attention was investigated using the KITAP task *“The Owls”* and the TAP task *“Divided Attention.”* These sub-tests were intended primarily to assess divided attention skills and comprised the managing of two modalities (visual and auditory). An adaptation was administered to assess selective auditory skills by using the auditory component only. In this adaptation, children listened to an auditory sequence during which two sounds were presented one at a time in regular alternation. Children had to press a reaction key as quickly as possible each time they detected an irregularity in the sequence (the same sound twice consecutively). The number of correct responses and the median time reaction were used as dependent variables.

##### Divided attention

Divided attention was assessed using the sub-test of the KITAP “*The Owls*” and the TAP task “*Divided Attention*”. These tasks were used as a dual measure to assess children’s ability to divide attentional resources between two perceptual modalities (visual and auditory). In the child version, children were required to press a response key as quickly as possible each time they detected an irregularity in the auditory sequence (two identical consecutive sounds as in the auditory task). In the visual modality, the child had to press a key as soon as possible each time an owl closed its eyes. In the adult version, the visual modality was composed of moving crosses; the children had to push when the crosses formed a square, while the auditory component was the same as in the auditory task. The number of correct responses and median reaction times were used as dependent variables.

##### Cognitive flexibility

Cognitive flexibility was measured by the KITAP task “*The Dragons’ House*” or the TAP task “*Letter-Number Alternation.*” In the children’s version, a green dragon and a blue dragon were presented randomly on each side of the computer screen. Children were required to alternate between the two dragons as fast as possible by pressing a response key corresponding to the side on which the target dragon was located on the screen. The side on which the target would appear was unpredictable. The adult version combined letters and numbers. The participant had to react on the number and the letter alternatively by pressing the right reaction key (in front of the target). The number of correct responses and the median reaction times served as the dependent variables.

##### Working memory

Working memory was measured by the subtest “backward digit span” of the Wechsler Intelligence Scale for Children-Fourth Edition (WISC-IV). This task was added to the protocol used by Nicolay and Poncelet (2013a, 2015) and Barbu et al. (2019) to evaluate the working memory performance of the immersed children, as this function seems to be “boosted” by the CLIL environment. Participants heard a digit sequence and were required to repeat it in reverse order. The sequences got progressively longer, ranging from a maximum of two to eight digits. The span (higher number of digits correctly repeated) was used as the dependent variable.

#### Ethics Statement

Each pupil participated voluntarily and parental consent was obtained. The study received approval from the Ethics Committee of the Faculty of Psychology, Speech Therapy and Education Sciences from the University of Liège.

#### General Procedure

The children performed the different tasks over a set of two sessions (approximately 40 min per session). The interval between the two sessions did not exceed three weeks. The tasks were administered in the same order for each child. We began with an easy task to put the child at ease, to continue with more demanding tasks (attentional and executive tasks), and to finish with more familiar tasks as more school like activities. We applied the alerting, working memory, selective auditory attention, non-verbal intelligence and L2 lexical tasks during the first session, followed by divided attention, cognitive flexibility and verbal intelligence tasks during the second session. Children were tested individually in a quiet room in their respective schools during the second semester of the school year (from February to April). All children were tested during the morning to avoid fatigability.

## Results

### Statistical Analysis

*T*-test and Chi-square tests were used to control the matching of the immersed and non-immersed groups in terms of gender, age, SES, L1 lexical level, non-verbal reasoning, and video game, music, and sport practice. We compared immersed and non-immersed groups in grade 1, grade 2, grade 3, and grade 6 in terms of attentional and executive performances. Analyses were conducted separately for the children in grades 1, 2, and 3 vs those in grade 6 because, as a reminder, we used two versions of the battery (child and adult version) to evaluate attentional and executive functions of these children. Concerning the three first grades, a series of two-way analysis of variance (ANOVA) was conducted to determine whether a difference would be found with school progression (time: grades 1, 2, 3) according to the group (immersed or non-immersed) in the different cognitive measures (alerting, selective auditory attention, divided attention, cognitive flexibility, and working memory). Concerning sixth grade, because the two groups were not matched on gender, we used a series of unique ANCOVA to determine whether a difference would be found according to the group (immersed or non-immersed) in terms of the different cognitive measures applied (alerting, selective auditory attention, divided attention, cognitive flexibility, and working memory).

Given issues concerning inferential statistics related to p-values, the null hypothesis, and statistical power (Wagenmakers, 2007; Wagenmakers et al., 2015), we also employed Bayesian analysis of variance (ANOVA) (Love et al., 2015^1^) to compare immersed and non-immersed on the different measures applied. The Bayesian factor (BF) given by these analyses reflects the likelihood ratio of the null model (null hypothesis, BF01) relative to the effect of interest (alternative hypothesis, BF10). It is generally acknowledged that it should considered that a Bayesian factor of 1 provides no evidence, 1 < BF > 3 provides anecdotal evidence, a BF greater than 3 provides moderate evidence, a Bayesian factor over 10 provides strong evidence, and a Bayesian factor higher than 30 provides very strong evidence (Lee and Wagenmakers, 2014). All the Bayesian analyses were performed using JASP (JASP Team, 2017) and we used default Cauchy prior distribution parameters as implemented in JASP (Version 0.8.5.1).

### Background Measures

There were no significant differences within each grade between the immersed and non-immersed groups on age, L1 lexical, non-verbal reasoning abilities (see Table 2), or SES, gender, and extra-scholar activities (see Table 3) except in sixth grade for gender. Given that the immersed and non-immersed children in grade 6 were not match on gender, we conducted an ANCOVA to control for this factor in further analysis.

### Attentional and Executive Measures in the Different Groups

**FIGURE 1:**
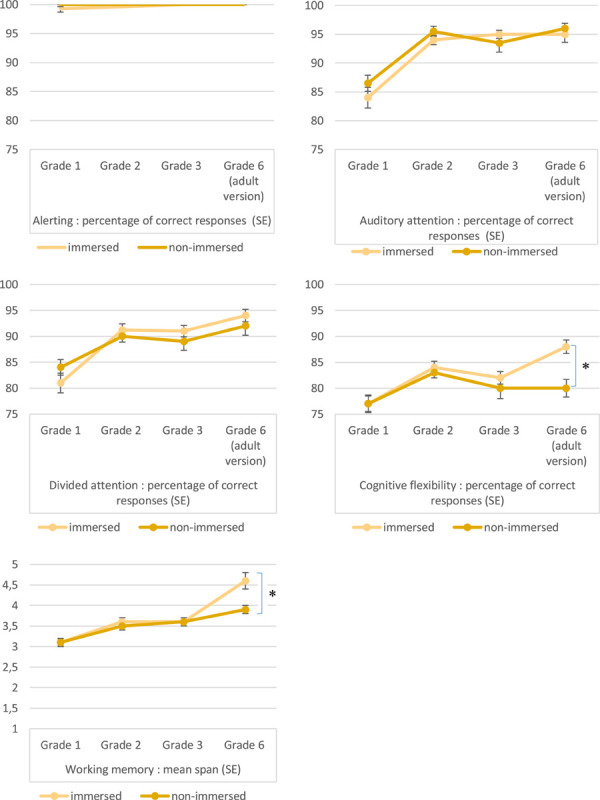
Linear graphs of percentage of correct responses in each grade group and the Standard Error (SE) for alerting, auditory attention, divided attention, cognitive flexibility tasks and mean span (SE) in each grade group for the working memory task.

At the beginning of the schooling, the results revealed an unsurprising significant main effect of grade for most of the cognitive functions measured (some showed a ceiling effect for correct responses). These results suggest that our measures are valid, with the children becoming better on tasks with age. The descriptive statistics in terms of median reaction times and correct responses concerning attentional and executive tasks are detailed below in Table 5. As the tasks used were different, we presented the results from grade 1 to grade 3 and those of grade 6 separately. The evolution of the performances at the different grades are represented in the linear graphs of the Figure 1 in which we convert the correct responses in percentage of correct responses to render the comparison across grades possible, except for working memory in which the raw score is presented (as percentage of correct responses cannot be calculated).

**TABLE 5:** Immersion characteristics and Dutch lexical acquisition for immersed children in each grade.

	**Grade 1 (*n* = 53)**	**Grade 2 (*n* = 53)**		**Grade 3 (*n* = 51)**	**Grade 6 (*n* = 39)**
**Mean of correct responses (SD)**					
Scholar productive vocabulary in L2 Max = 135	44.0 (17.5)	57.5 (22.3)	Dutch adaptation of Expressive One-Word Picture Vocabulary Test Max = 170	13.5 (8.6)	39.8 (11.8)
Scholar receptive vocabulary in L2 Max = 135	98.3 (15.3)	110.1 (14.2)	Dutch version of the Peabody Picture Vocabulary Test Max = 150	67.1 (21.8) Lexical age: 4;9 years	97.4 (17.5) Lexical age: 7;6 years

#### Alerting

##### Grades 1 to 3 (Kitap)

Concerning correct responses, an inferential two-way analysis of variance revealed no effect of Group (immersed vs. non-immersed) (*F* (1,307) = 2.58, *p* = *0.10*, η*p*^2^ < 0.01), no effect of Time (grade 1-2-3) (*F* (2,307) = 1.82, *p* = *0.16*, η*p*^2^ = 0.01), and no significant interaction (*F* (2,307) = 1.82, *p* = *0.16*, η*p*^2^ = 0.01). Bayesian two-way analysis of variance supported the null hypothesis concerning the Group effect for alerting (*BF_10_* = 0.4; *BF*_01_ = 2.3, error% 3.388e-6).

Concerning reaction times, inferential two-way analysis of variance showed no effect of Group (*F* (1,307) = 0.43, *p* = *0.51*, η*p*^2^ < 0.01) and an effect of Time (*F* (2, 307) = 15.55, *p* < 0.001, η*p*^2^ = 0.09) but no Time^∗^Group interaction (*F* (2,307) = 0.51, *p* = *0.59*, η*p*^2^ < 0.01). Bayesian double ANOVA decisively supported the null hypothesis concerning the Group effect for alerting (*BF_10_* = 0.1, *BF*_01_ = 6.9, error% 1.479e-5).

##### Grade 6 (TAP)

Concerning correct responses, we were confronted with a ceiling effect that did not permit further analysis.

Concerning reaction times, inferential ANCOVA controlling for gender (because the two groups were not matched on this variable) revealed no effect of Group (*F* (1,75) = 0.03, *p* = *0.85*, η*p*^2^ < 0.01). Bayesian ANCOVA supported the null hypothesis concerning Group effect (*BF*_10_ = 0.2; *BF*_01_ = 4.2, error% 0.019).

#### Selective Auditory Attention

##### Grades 1 to 3 (Kitap)

Concerning correct responses, inferential two-way analysis of variance showed no effect of Group (*F* (1,307) = 0.56, *p* = 0.45, η*p*^2^ < 0.01). There was an effect of Time (*F* (2,307) = 32.26, *p* < 0.001, η*p^2^* = 0.17) but no interaction (*F* (2,307) = 1.01, *p* = 0.36, η*p*^2^ < 0.01).

Bayesian two-way analysis of variance ANOVA decisively supported the null hypothesis concerning Group effect for auditory selective attention (*BF*_10_ = 0.1; *BF*_01_ = 6.2, error% 1.307e-5).

Concerning reaction times, inferential two-way analysis of variance showed that no effect of Group was found (*F* (1,307) = 0.34, *p* = 0.55, η*p*^2^ < 0.01). As expected, we found an effect of Time (*F* (2,307) = 27.47, *p* < 0.001, η*p*^2^ = 0.15) but there was no interaction (*F* (2,307) = 0.77, *p* = 0.46, η*p*^2^ < 0.01). Bayesian two-way analysis of variance decisively supported the null hypothesis concerning Group effect (*BF*_10_ = 0.1; *BF _01_* = 7.2, error% 1.569e-5).

##### Grade 6 (TAP)

Concerning correct responses, inferential ANCOVA controlling for gender showed no effect of Group (*F* (1,75) = 0.01, *p* = *0.90*, η*p*^2^ < 0.01). Bayesian ANCOVA supported the null hypothesis concerning Group effect on correct responses (*BF_10_* = 0.2; *BF*_01_ = 4.1, error% 0.019).

Concerning reaction times, inferential ANCOVA controlling for gender showed no effect of Group (*F* (1,75) = 0.76, *p* = 0.38, η*p*^2^ = 0.01). Bayesian ANCOVA supported the null hypothesis concerning Group effect on reaction times (*BF_10_* = 0.2; *BF*_01_ = 3.8, error% 0.019).

#### Divided Auditory Attention

##### Grades 1 to 3 (Kitap)

Concerning correct responses, inferential two-way analysis of variance showed no effect of Group (*F* (1,307) = 0.57, *p* = *0.45*, η*p*^2^ < 0.01), an effect of Time (*F* (2,307) = 32.26, *p* = 0.11, ηp^2^ = 0.17), and no Time^∗^Group interaction *(F* (2,307) = 1.01, *p* = *0.36*, η*p*^2^ < 0.01). Bayesian two-way analysis of variance decisively supported the null hypothesis concerning the Group effect for divided attention (*BF*_10_ = 0.1; *BF*_01_ = 8.0, error% 1.100e-5).

Concerning reaction times, again, inferential two-way analysis of variance showed no effect of Group (*F* (1,307) = 0.98, *p* = 0.32, η*p*^2^ < 0.01). An effect of Time (*F* (2,307) = 17.22, *p* < 0.001, η*p^2^* = 0.10) and a Time^∗^Group interaction (*F* (2,307) = 4.04, *p* = 0.01, η*p*^2^ = 0.02) were found. Planned comparisons between the two groups inside each grade revealed that the immersed group responded more slowly in first grade (*F* (1,307) = 4.72, *p* = *0.03*). In second (*F* (1,307) = 2.84, *p* = *0.09*) and third grades (*F* (1, 307) = 1.41, *p* = *0.23*), the performances of the two groups did not differ significantly. Bayesian two-way analysis of variance decisively supported the null hypothesis concerning Group effect (*BF10* = 0.1; *BF01* = 5.5, error% 1.793e-5). A Bayesian comparison could not support the significant difference found in first grade (*BF_10_* = 1.4, *BF*_01_ = 0.6) with inferential planned comparisons. The Time model was the model with the highest BF (*BF*_10_ = 101910.2) over the models including Group^∗^Time interactions (*BF*_10_ = 38345.6), which confirms that it is Time that mostly explain the evolution of the performance in reaction times.

##### Grade 6 (TAP)

Concerning correct responses, inferential ANCOVA controlling for gender showed no effect of Group (*F* (1,75) = 1.20, *p* = 0.27, η*p*^2^ = 0.01). Concerning reaction times, we found no effect of Group (*F* (1,75) = 0.10, *p* = 0.75, η*p*^2^ < 0.01). Bayesian ANCOVA supported the null hypothesis concerning Group effect for correct responses (*BF*_10_ = 0.3; *BF*_01_ = 3.2, error% 0.019) and reaction times (*BF*_10_ = 0.2; *BF*_01_ = 4.2, error% 0.019).

#### Cognitive Flexibility

##### Grades 1 to 3 (Kitap)

Concerning correct responses, inferential two-way analysis of variance showed no effect of Group (*F* (1, 302) = 0.94, *p* = *0.33*, η*p*^2^ < 0.01) and an effect of Time (*F* (2, 302) = 9.66, *p* < *0.001*, η*p*^2^ = 0.05). No interaction (*F* (2, 302) = 0.26, *p* = *0.77*, η*p*^2^ < 0.01) was found. Bayesian two-way analysis of variance supported the null hypothesis concerning Group effect (*BF*_10_ = 0.1; *BF*_01_ = 5.4, error% 1.094e-5).

Concerning reaction times, inferential two-way analysis of variance showed a Group effect (*F* (1, 307) = 4.09, *p* = 0.04, ηp^2^ = 0.01). Again, an unsurprisingly effect of Time was found (*F* (2, 307) = 43.42, *p* < 0.001, η*p*^2^ = 0.22). Furthermore, no interaction was found (*F* (2, 307 = 1.35, *p* = *0.25*, η*p*^2^ < 0.01). Planned comparisons between the two groups in each grade revealed that the immersed group responded more slowly in first grade (*F* (1, 307) = 5.91, *p* = *0.01*) but no difference was found in second (*F* (1, 307) = 1.05, *p* = *0.30*) and third grades (*F* (1, 307) = 0.00, *p* = *0.93*). Concerning Bayesian two-way analysis of variance, the direction of the results is less clear (*BF_10_* = 0.5; *BF*_01_ = 1.8, error% 2.538e-6); the Bayesian factor stems for an anecdotal support (BF between 1 and 3) of the null hypothesis.

##### Grade 6 (TAP)

Inferential ANCOVA, concerning correct responses, showed an effect of Group (*F* (1,75) = 7.94, *p* < 0.01, η*p*^2^ = 0.09) in favor of the immersed group. Bayesian ANCOVA supports the alternative hypothesis (*BF_10_* = 30.4; *BF*_01_ = 0.03, error% 2.239e-4).

Concerning reaction times, the results revealed no Group effect (*F* (1, 75) = 0.31, *p* = 0.57, η*p*^2^ < 0.01). Bayesian ANCOVA supports the null hypothesis (*BF_10_* = 0.3; *BF_01_* = 3.1, error% 0.018).

#### Working Memory (Span)

##### Grades 1 to 3

Inferential two-way analysis of variance revealed no effect of Group (*F* (1, 302) = 1.16), *p* = *0.28*, η*p*^2^ < 0.01) and an effect of Time (*F* (2, 302) = 14, 84, *p* < 0.001, η*p*^2^ = 0.08). No interaction (*F* (2, 302) = 0.49, *p* = *0.60*, η*p*^2^ < 0.01) was found. Bayesian two-way analysis of variance decisively supported the null hypothesis concerning Group effect for working memory (*BF*_10_ = 0.2; *BF*_01_ = 5.0, error% 5.680e -6).

Grade 6

ANCOVA showed an effect of Group (*F* (1.75) = 9.21, *p* < 0.01, η*p*^2^ = 0.10) in favor of the immersed group. Bayesian ANCOVA supports the alternative hypothesis (*BF*_10_ = 3.7; *BF*_01_ = 0.2, error% 0.002).

To resume, based on Bayesian statistics, no difference between the groups (immersed vs. non-immersed) was found in grades 1, 2, and 3. In grade 6, the immersed group outperformed the non-immersed group in the cognitive flexibility (correct responses) task and the working memory task.

### Dutch Lexical Acquisition for Immersed Children in Each Grade

Table 4 describes the results of the Dutch vocabulary tasks. We used the same tasks to evaluate children in first and second grades but used another task in third and sixth grades. Comparisons showed a significant difference between first and second grades in comprehension (*F* (1, 102) = 21.37, *p* < 0.0001, η^2^ = 0.17) as well as production (*F* (1, 102) = 13.1, *p* < 0.001, η^2^ = 0.11) and between third and sixth grades in comprehension (*F* (1, 79) = 165, *p* < 0.0001, η^2^ = 0.69) and production (*F* (1, 79) = 415.3, *p* < 0.0001, η^2^ = 0.84). The test used in third and sixth grades was a standardized test so that we could compare the performances with monolinguals. Concerning the L2 vocabulary level, it is interesting to note that the children in grade 3 (105 months ± 4 or approximately 8 1/2 years old) obtained an average level of proficiency in lexical comprehension comparable with 4; 9 years old Dutch monolingual children. Moreover, in grade 6 (139 months ± 3 or approximately 11 1/2 years old), on average, the Dutch immersed group performed as did 7; 6 years old Dutch non-immersed children on the same task.

## General Discussion

The present study explored AEF performance in children enrolled in Dutch L2 learning programs at different moments of primary schooling (i.e., first, second, third, and sixth grades). The main aim was to determine whether the length of time spent in CLIL could play a role in the emergence of cognitive advantages using the same tasks having shown an advantage in previous studies conducted in a same CLIL context with English as L2.

Globally, regarding the influence of time spent in immersion, our results contradict the ones of studies conducted with English immersed children. In the present study, conducted with Dutch immersed children, time spent in immersion seems to be an important factor as the AEF advantages emerge only at the end of CLIL program, after 6 years of L2 exposition. During the first three years of CLIL schooling, we did not find an advantage in any tasks proposed. In contrast, at the end of the CLIL program, we found an advantage in favor of the immersed group on two tasks evaluating cognitive flexibility and working memory. Contrarily, in previous studies conducted with English immersed children, advantages emerge from first to third grade and are not present at the end of the primary schooling. We will discuss below, the impact of the time spent in the immersion program on AEF performances and some of the factors that could explain the differences of outcomes among the studies conducted until now.

### Impact of the Time Spent in the Immersion Program on AEF Performances

During the first years of immersion in Dutch, no cognitive advantage was found in the present study. Among the studies evaluating the same AEF as those evaluated in the present study and conducted in first grade (*or after one year of immersion*) but using different tasks to evaluate AEF, Woumans et al. (2016) found no advantages in either attentional or executive tasks (verbal fluency task, Simon task, ANT) in French-speaking children learning Dutch as L2. Kalashnikova and Mattock (2014), contrarily, found an advantage in cognitive flexibility (DCCS) in English-speaking children learning Welsh. Note that in this study, the children lived in a Welsh-English bilingual community and were surrounded by bilingual adults. Barbu et al. (2019), with the same tasks as ours, found no advantages for alerting, divided attention, and cognitive flexibility (KITAP battery) in French-speaking children immersed in English. However, they found an advantage in the auditory selective attention task. Further, Puric et al. (2017) did not find an advantage in the cognitive flexibility tasks (local-global task and color-shape task) in Serbian-speaking children learning English or German as L2 but did find an advantage in working memory tasks (counting recall task and backward digit span task). Note that the amount of time spent in immersion of these children was high (5h/day).

Among the studies evaluating the same AEF but using different tasks as those used in the present study and evaluating children in second grade (*or after two years of immersion)*, Kaushanskaya et al. (2014) also did not find an advantage in a cognitive flexibility task (DCCS) in English-speaking children immersed in Spanish. However, these authors found an advantage in a task evaluating working memory (listening-span task) for immersed children. Note that these children were immersed for 90% of their school time, which is far more than our immersed sample (50-75% of L2 exposition during class time).

Among the studies evaluating the same AEF in third grade (*or after three years of immersion*), all used the same tasks as those used in the present study. Nicolay and Poncelet (2013a, 2015), in contrast to our study, found advantages in alerting, selective auditory attention, divided attention, and cognitive flexibility tasks (KITAP battery) in French-speaking children learning English as L2.

In sum, for the first three years, the results are inconsistent across the different studies and it is difficult to interpret the reason for these inconsistencies, as in most of the studies, the amount of immersion, the languages at stake, and the tasks vary. Moreover, few of these studies control for all the variables known to influence AEF. Nevertheless, even in comparison to the studies of Barbu et al. (2019) and Nicolay and Poncelet (2013a, 2015), in which most of the confounding variables are controlled, and in which the tasks used and the CLIL context frequented by the children are similar, the results are not convergent. However, there is one difference between these two studies and the present one: the language of immersion (L2), which was English in Barbu et al. (2019) and Nicolay and Poncelet (2013a, 2015) and which was Dutch in the present one. Thus, the CLIL impact on AEF performances could vary depending on the L2 learned.

In sixth grade, we do not find any advantages for alerting, selective auditory attention, and divided attention tasks in favor of the immersed children. In contrast, we found an advantage in the immersed group for cognitive flexibility and working memory tasks. As a reminder, the non-immersed children received L2 instruction during the two last years of schooling (grades 5 and 6), but this does not seem to have had an impact on their performance. In any case, the CLIL context impact seems to be more important than that of a traditional foreign language course of two hours per week. In contrast, Simonis et al. (2019) and Simonis (2019), respectively, did not find an executive advantage on tasks evaluating cognitive flexibility or inhibition (DCCS, Simon task, ANT) after 5 years and an attentional advantage (different attentional tasks from the TAP battery) after 6 years of immersion in French-speaking children learning Dutch or English as L2. Otherwise, they did not evaluate working memory. Nicolay (2012) further did not find an advantage in terms of alerting, selective auditory attention, divided attention tasks, or cognitive flexibility in sixth grade, while they used the same task as in the present study in French-speaking children learning English as L2. Again, this difference in outcomes could be linked to the immersion language (L2). Note that the significant difference revealed in the present study concerned accuracy scores and not reaction times in cognitive flexibility. In Nicolay (2012), only reaction time data were presented because accuracy scores were very high on each task, according to the authors. Working memory was not evaluated in Nicolay (2012).

Globally, these results suggest that the CLIL context could confer cognitive advantages at different moments of the schooling, which could vary depending on the second language learned. Indeed, in children immersed in Dutch, we found no cognitive advantages in first, second, and third grades but we found an advantage in sixth grade. Contrariwise, in children immersed in English, in previous studies using the same tasks, an advantage in first and third grades was found but not in sixth grade. Does Dutch take more time to master and, as a consequence, does its learning provide cognitive advantages later in the cursus? Or could the linguistic characteristics and/or status of the second language learned influence which AEF and when it could be enhanced in the CLIL cursus? Finally, the inconsistency of the results could also be because certain studies did not sufficiently control for the samples’ characteristics.

We will consider these hypotheses below.

### Factors That Could Explain the Differences in Performance in the Studies

#### Characteristics of the Immersed and Non-immersed Samples

In the present study, we try to control for different factors that were believed to influence the attentional and executive functions: video game, music playing, and sport practice, in addition to age, grade and SES, non-verbal reasoning, and lexical L1 level. However, certain studies showing an early advantage did not control for these same factors. For example, Kalashnikova and Mattock (2014), showing an advantage in first grade, controlled only for age and L1 receptive vocabulary. Although the studies of Nicolay and Poncelet (2013a, 2015) controlled for many factors, showing advantages in favor of immersed children after 3 years of immersion, the studies did not control for video game use, music playing, and sport practice. Note, however, that Barbu et al. (2019) used the same control variables as those in the present study and showed a slight difference after one year of English immersion on selective auditory attention. The outcomes of the study of Barbu et al. (2019) and the present one suggest that even while closely controlling for confounding variables, a cognitive advantage could be highlighted in the CLIL environment. The only difference between the study of Barbu et al. (2019) and the present study is the second language learned, respectively, English and Dutch.

#### The Linguistic Characteristics of the Languages at Stake

Among highly similar CLIL studies, like in the Belgian ones, the AEF advantage appears later in Dutch than it does in English as L2. One explanation could come from the different linguistic structures of the languages at stake. Given the differences between the English and Dutch languages on the lexical, orthographic (opaque orthographies), and syntactic levels, the learning of these languages for a French-speaking child could be related to different cognitive solicitations at different moments of the L2 learning. The English-French pair is indeed more similar than the Dutch-French one. At the lexical level, English is closer to the French language than to Dutch given their shared history, which has led to reciprocal lexical loans (as for example, compensation, double, impulsion, membrane, sentimental, volume; Walter, 2001). At the orthographic level, English and French use two opaque orthographic codes, while Dutch has a highly transparent orthographic code. At the syntactic level, Dutch structure is said to be head-final, whereas the English and French structures are head-initial. For example, Dutch has a subject-object-verb (SOV-like) underlying structure (Koster, 1975). In Dutch, some verbal forms are placed at the end of the sentence. This is the case with the infinitives (e.g., De kinderen moeten fruit *eten*; Les enfants doivent *manger* des fruits, which means, The kids have *to eat* fruits) and past participles (e.g., Ik heb een fruit *gegeten*; J’ai *mangé* un fruit, which means, I have *eaten* a fruit). Moreover, in sub-clauses, all verbal forms are rejected at the end of the sentence (e.g., Ik zie de kat die de hond *aanvalt*; Je vois le chien qui *attaque* le chat, which means, I see the dog that *attacks* the cat). Because the verb can be regarded as the head of the predicate, Dutch structure is said to be head-final (the head of the phrase—that is to say, the verb—is in the final position), whereas the English and French structures are head-initial (the head of the phrase—that is to say, the verb—is in initial position). Thus, these different characteristics of the languages at stake could indicate that Dutch, which could be more complex to learn for French-speaking children than English, takes more time to fully master. Consequently, AEF advantages such as cognitive flexibility could appear later in Dutch-immersed children. Note, however, that the children immersed in Dutch from the present study have a similar L2 lexical level (see Table 5) as children immersed in English from previous studies using the same L2 lexical tasks. Actually, children in first grade from Barbu et al. (2019) obtained a receptive vocabulary score of 92.30 ± 22.50 and a productive vocabulary score of 40.69 ± 21.90. In Nicolay and Poncelet (2013a), children in third grade attained an L2 receptive lexical age of 4; 9 years (Peabody Picture Vocabulary Test). In Nicolay (2012), children in sixth grade attained an L2 receptive lexical age of 8;5-year-old (Peabody Picture Vocabulary Test). Nevertheless, learning a second language is not limited to the lexical level. Also, the other linguistic levels—in particular, the syntactic one—could be more complex to learn in Dutch than in English given that it differs in its structure from French and English (SVO vs. SOV). Moreover, while English could be easier to master than Dutch on the syntactic level, it is omnipresent in the children’s environment (e.g., video games, music, social media) in Belgium as in numerous countries of the world. This could contribute to the more rapid mastery of L2 when learning English (De Wilde et al., 2019). Thus, children learning English as L2 could more quickly show a higher rate of switching behaviors between L1 and L2, as a certain level of L2 and a number of exposition opportunities are required to lead to switching behaviors. This switching behavior has been related to better cognitive flexibility abilities in bilingual adult studies (e.g., Barbu et al., 2018; Barbu et al., 2020). These authors compared two groups of highly proficient bilinguals and found an advantage in the cognitive flexibility task in favor of the group that presented a high (vs. low) rate of switching behaviors in daily life. Moreover, López-Penadés et al. (2020) showed, in early bilingual adults, that frequent switching to the second language was associated with more efficient executive processing, such as a better shifting ability (beyond the age of second language acquisition and language proficiency). Thus, if children immersed in English switch more frequently, this could better train their cognitive flexibility. This could explain why the advantage in the cognitive flexibility task appears sooner in their schooling than in children immersed in Dutch.

Finally, concerning the advantage in terms of working memory, some studies showed that different syntactic structures could induce some particularities in terms of the way we maintain information. For example, Amici et al. (2019) showed that the syntax and word order of a language predicts the way we remember verbal and non-verbal stimuli in working memory tasks. In their study, a series of stimuli were presented to participants who spoke either a language with a head-final syntactic structure (e.g., Japanese, Korean…) or a language with a head-initial structure (e.g., Italian, Khmer …). The participants were required to solve a distracting task, and then to recall the stimuli in the same order as they were presented. Head-final speakers were better at maintaining initial stimuli and head-initial speakers were better at maintaining final stimuli of the sequence. Compared to Amici’s study, the working memory of French-speaking children learning Dutch—which is a head-final language—could be solicited differently. This additional solicitation could, in turn, lead to an enhancement of its capacity at the end of the CLIL schooling, when children are more likely to be exposed to increasingly long and complex sentences in their L2. To confirm the role of the syntactic structure in the working memory advantage, it would be necessary to conduct the same study with French-speaking children immersed in English, evaluating working memory at the beginning and at the end of the CLIL schooling. These children should not show an advantage, as French and English are both head-initial languages.

Hansen et al. (2016) also evaluated working memory performances at the end of the CLIL primary schooling in Spanish-speaking children learning English—two head-initial languages—and did not show an advantage. This seems to correspond to our hypothesis of the necessary role of syntactic structure. However, Hansen et al. (2016) also evaluated working memory in children at the beginning of CLIL schooling and found an advantage in these children. Similarly, Puric et al. (2017) and Kaushanskaya et al. (2014) found a working memory advantage at the beginning of CLIL schooling. Other factors could, thus, intervene in the results concerning working memory. However, the outcomes of these studies should be carefully compared to ours, as they included very small samples of children immersed at a high rate (near 100%) from the start of the CLIL schooling. Moreover, the variables used to match the groups and the languages at stake were not the same as those in the present study.

#### Status of the Second Language

In addition to the particular structure of Dutch, note that this language is not as ubiquitous as English in daily life (e.g., music, video games). The omnipresence of English could render the learning of this language more attractive to children, which, in turn, could lead them to be more motivated to learn it. Being motivated to learn the second language (Pintrich, 1999; Lasagabaster, 2011; Lasagabaster et al., 2014; Gardner and Yung, 2015; Dörnyei, 2019) and being more exposed to this L2 out of school, in informal contexts (De Wilde et al., 2019) are factors known to enhance L2 learning. In the second language learning literature, motivational and affective factors have already been pointed out. Some authors have, for example, shown that Dutch learning, in comparison to English learning, is less attractive to children in terms of enjoyment and is more likely to provoke anxiety in the CLIL context (De Smet et al., 2018; see also Mettewie, 2004, 2015). Consequently, the children could need more time to master the L2 when learning Dutch and, in turn, the cognitive advantages could appear later. Indeed, a certain level of L2 is necessary to switch from one language to another. In sum, we could hypothesize that learning Dutch, as it seems to be less attractive, could result in slower development. This could explain the attentional and executive differences of performance or, at least, the different timing, wherein advantages emerge, which have been found in the studies evaluating French-speaking children learning Dutch or English.

## Conclusion

To conclude, our study comparing children following a bilingual education in Dutch since 1, 2, 3, and 6 years, and control children, on attentional and executive tasks, seems to provide evidence of an advantage in terms of cognitive flexibility and working memory in Dutch primary immersion in the sixth grade but not in the first, second and third grades. We used tasks already known to show an advantage in children learning English as L2 in the CLIL context (Nicolay and Poncelet, 2013a, 2015; Barbu et al., 2019) but did not replicate the same results with children immersed in Dutch, as the advantages appeared later and only in certain cognitive functions. In the future, it would be interesting to compare French-speaking children in fourth and fifth grades learning English or Dutch with the same tasks as those used in the present study to better understand how AEF progresses between third and sixth grades in the function of the languages at stake. A study evaluating working memory in French-speaking children learning English (with an SVO structure like in French) would also be interesting for purposes of determining the role of the L2 characteristics in the WM advantage. Other studies would try to replicate the results with the same tasks but with a more robust design, like a longitudinal one, to ensure that the positive results could not be explained by sample bias. This design could also provide more information about the sustainability of the advantages in time. To more precisely assess the L2 mastery of immersed children, it would also be required to assess not only the lexical level of mastery but also the syntactic level. Comparing different L1-L2 pairs could also clarify the specific impact of the differences in terms of status, syntactic, or other linguistic characteristics, between the languages at stake, on attentional and executive functions. Finally, using a more precise measure to evaluate extra scholar activities could also help to better control these variables. To conclude, the emergence of cognitive advantages may vary depending on the characteristics of the second language learned. This variable of mother tongue and second language characteristics should be considered in further studies.

## Data Availability Statement

The raw data supporting the conclusions of this article will be made available by the authors, without undue reservation.

## Ethics Statement

The studies involving human participants were reviewed and approved by the Ethics Committee of the Faculty of Psychology, Speech Therapy and Education Sciences from the University of Liège. Written informed consent to participate in this study was provided by the participants’ legal guardian/next of kin.

## Author Contributions

SG and MP designed the study and wrote the manuscript with help from CB. SG collected the data with help from CB, and also performed the analyses. MP contributed to designing the analyses. All authors contributed to the article and approved the submitted version.

## Conflict of Interest

The authors declare that the research was conducted in the absence of any commercial or financial relationships that could be construed as a potential conflict of interest.

## Footnotes

1 https://jasp-stats.org/
